# Agreement between child self-report and parent-proxy report for functioning in pediatric chronic pain

**DOI:** 10.1186/s41687-024-00774-0

**Published:** 2024-08-09

**Authors:** Joan W. Hanania, Jessica Edwards George, Christie Rizzo, Justin Manjourides, Laura Goldstein

**Affiliations:** 1 Bouvé College of Health Sciences, Northeastern University, Boston, MA USA; 2 Department of Child and Adolescent Psychiatry, Boston Medical Center, Boston, MA USA; 3 Department of Psychosocial Oncology and Palliative Care, Dana-Farber/Boston Children’s Cancer and Blood Disorders Center, Boston, MA USA

**Keywords:** Pediatric chronic pain, Coping and adaptation, Child self-report and parent-proxy report, Child-parent agreement, Pain-related functional disability

## Abstract

**Purpose:**

Accurate assessment of chronic pain and functional disability in children and adolescents is imperative for guiding pain management interventions. Parents have multifaceted roles in their child’s pain experience and frequently provide parent-proxy reports of pain-related functioning. However, cross-informant variance is often observed with limited understanding of contributing factors. This study aims to examine the degree of alignment between child and parent-proxy reports for Patient-Reported Outcomes Measurement Information System (PROMIS) pain interference domain among children with chronic pain and to identify factors associated with improved child-parent agreement.

**Methods:**

This study includes a sample of 127 youth (66.1% female) with mixed etiology chronic pain, ranging in age from 8 to 17 (M = 12.24; SD = 1.598), and their parent. Data was collected at an interdisciplinary pediatric pain clinic and online peer support groups. Measures of demographic, pain intensity, and functioning were collected.

**Results:**

Means of parent-proxy reports were significantly lower than child self-reports on the PROMIS (*p* < 0.05). A statistically significant association between child’s pain intensity (β = 0.953, *P* < 0.05) and the difference between child self-reported and parent-proxy reported PROMIS functional interference scores was found.

**Conclusion:**

Parents underestimated pain-related functional disability relative to children’s self-reports. The difference between the paired child self-report and parent-proxy report of functional disability was significantly associated with greater child self-reported pain intensity. Although parent-proxy reports in pediatric chronic pain is often used in research and practice, findings underscore the importance of incorporating child and adolescent self-report, when possible, to comprehensively capture the child’s pain experience and best inform clinical interventions.

## Introduction

Pediatric chronic pain refers to any prolonged pain that lasts longer than the expected healing time or recurrent pain that occurs at least three times over a period of three months [1]. Pediatric chronic pain is associated with negative social and psychological sequelae [2, 3]. A plethora of research documents the link between chronic pain and functional disability [2, 4–5]. Given the complexity of chronic pain, the functioning of children and adolescents across multiple domains is impacted by the recurrent experience of chronic pain. Research suggests that 5–8% of youth with chronic pain will experience decreased functioning and pain-related functional disability [2–4, 6, 7]. Pediatric chronic pain has been further associated with negative school attendance and decreased levels of social and physical functioning [5, 8, 9]. Given the pervasive impact of chronic pain on functioning, pediatric chronic pain assessment and treatment often targets physical, psychological, academic, behavioral, and social domains [10, 11].

Parents play a key role in the child’s experience of pain and functional disability [2, 9, 12, 13]. Integrative models of pediatric chronic pain [2] and biobehavioral models [14], support the inclusion of biological, parental, and environmental factors contributing to the pain experience in children and adolescents. Pediatric chronic pain and associated functional disability is experienced through and influenced by the child and adolescent’s multidimensional interpersonal interactions with their social environment.

In pediatric settings, parents and children provide reports of pain symptoms and associated functioning. Reliance on children and adolescents report of their own pain experience brings additional challenges related to developmental considerations and the utility of valid child self-report tools. Parent reports are, thus, readily integrated into symptom assessment and associated functioning across the pediatric chronic pain care continuum. Parents perceptions of the child and adolescent’s pain related outcomes impact the child’s pain experience, use of health care services, medical-decisions, treatment expectations, and goals of care [15]. Misalignment between child and parent perceptions around pain-related functional interference may contribute to undertreatment of symptoms or to overtreatment based on parental reports of perceived symptom burden. Research exploring pain experiences demonstrates how children and adolescents may feel misunderstood [16] and disbelieved [17, 18] particularly due to the fluctuating pain intensity and dynamic needs. Therefore, further examination of child-parent agreement on pain-related functional interference is needed.

Limited studies have focused on examining the level of agreement between child and parent’s perceptions of chronic pain and functioning. Previous studies yielded variable cross-informant agreement rates between child self-report and parent-proxy reports of pain intensity and functioning. Some studies showed parental underestimation of child’s pain-related functional disability [19–22], and others suggested that children provided lower ratings of pain intensity and overall well-being when compared to parental reports [23]. These inconsistencies may be attributed to sample and methodological differences in assessing pain intensity and pain-related functional outcomes.

Given the reported inconsistencies to date, further investigation to better characterize the degree of agreement between child and parent-proxy reports is warranted. Additionally, there is a growing need to delineate characteristics associated with the magnitude of difference between child self-report and parent-proxy report of pediatric pain-related functional disability. Therefore, the current study extends existing knowledge by examining the relationship between child self-report and parent-proxy reports of pain-related functional interference and identified unique factors associated with better child and parent-proxy congruence to better inform clinical practices in pain management settings.

## Method

### Participants

Children and adolescents with chronic pain and their parents and/or guardians participated in this study. Data was collected at an interdisciplinary pediatric pain clinic within an urban large public hospital setting in New England, as well as online through peer pain management support groups. Children and adolescents were aged 8 to 17 years with chronic pain of any etiology were eligible to participate in the study with parental consent. The child needed to be able to read and understand English and have no clinically significant cognitive and developmental impairment. Youth participants were ineligible to participate if they ty had major co-morbid medical illnesses (e.g., cerebrovascular and oncology) and/or were participating in psychological pain management treatment.

### Study design

All parental participants provided written informed consent and child participants provided assent. Participants completed a collection of standardized child self-report or parent-proxy measures. Data collection was managed by Research Electronic Data Capture (RedCap), a secure web application for building and managing online surveys and databases. The research study was approved by Institutional Review Board Committees at two local institutes including an academic university and an academic medical hospital. Each participating child-parent dyad received a $25 gift card in compensation for completing the study. All participants were assigned a unique identification number to protect the privacy and confidentiality of participants. All data materials with identifying information was saved securely in a locked cabinet at the participating hospital. All electronic information collected from participants was handled in compliance with the HIPPA privacy regulations.

### Measures

#### Sociodemographic and disease-related questionnaire

A brief parent-report demographic survey was developed to assess the child and parent’s demographic information including both close-ended and open-ended questions. Child and adolescent disease-related information, including the primary diagnosis of chronic pain, age of onset, other medical and psychiatric comorbidities, and psychological treatment received were collected.

#### Pain intensity

The Faces Pain Scale–Revised (FPS-R) [24] is a self-report measure used to assess pain intensity in children and adolescents. This measure consists of 6-line drawings of faces that present gradually increasing pain intensity ranging from *“No pain”* at the far left (scored 0) to *“Very much pain”* at the far right (scored 10). Respondents were asked to select the face that best reflect the intensity of their pain from a series of faces depicting varying pain intensity levels. The FPS-R scale has demonstrated adequate reliability, content validity, and construct validity for youth ages 4–16 years [25–27].

#### Functional disability

PROMIS pediatric and parent-proxy measure on pain interference, Short Form 8a [13] is an 8-item measure assessing perceived activity limitation and difficulties in physical and psychosocial functioning due to pain. Questions have 5 response categories, with higher scores indicating more pain interference. PROMIS Pediatric and parent proxy-report scores are converted into a common T-score distribution, with a mean of 50 (SD = 10), normed against the United States general population matched on sex, age, and race to the 2000 US Census [14, 28]. Findings support the feasibility, validity, and construct validity of the PROMIS Pain Interference measure [14, 28, 29]. Cronbach’s alpha estimate in the present sample was 0.88 suggesting internal reliability.

### Analytic strategy

The study employed a cross-sectional, multi-informant survey design. The child-parent agreement was assessed at the level of the individual according to intraclass correlation coefficient (ICC) and at the level of the group according to comparison of means. Bland-Altman plots were used to evaluate the tendency of parent-proxy reports to overestimate or underestimate pain related functional disability when compared to self-reports of children and adolescents with chronic pain.

A linear stepwise regression model was used to examine the influence of other factors including self-report pain intensity on the degree of agreement between raters. Specifically, the association among youth’s age in years, youth’s sex and race, self-report pain intensity, youth pain acceptance, parental pain acceptance, and parental psychological flexibility, and the difference between youth and parent reported PROMIS scores will be examined. A stepwise regression method is suggested for this analysis, with *P* < 0.05 entry criterion. The analyses will follow the recommended correlation values of 0.10 to 0.29 = small (weak), 0.30 to 0.49 = medium (moderate) and 0.50 to 1.0 = large (strong) were applied (Cohen, 1988), with a priori criterion value of 0.50 required for adequate youth-parent rating concordance.

Data was analyzed with SPSS Statistics software [30]. Two-tailed *P* < 0.05 indicated statistical significance.

## Results

### Child and parent-proxy characteristics

One hundred thirty-two youth and parent dyads consented to participate and enrolled in the study. Of those 132 participants, 5 dyads were excluded because they have not met inclusion criteria yielding a final sample of 127 child-parent dyad. The majority of the participants were recruited through several online peer support groups (*N* = 116, 91.3%), and the remaining participants completed the study following pediatric pain clinic visit.

Participants for this study were a sample of 127 youth (66.1% female) who were primarily White, non-Hispanic (*N* = 114, 89.8%), ranging in age from 8 to 17 (M = 12.24; SD = 1.598), and their parent or guardian (see Tables 1 and 2). The parent sample was composed of primarily White, non-Hispanic (*N* = 115, 89.9%) participants and included almost an equal number of participating mothers (*N* = 65, 51.18%) and fathers. Primary pain diagnoses included chronic abdominal pain (*N* = 38, 29.9%), musculoskeletal pain (*N* = 33, 26.0%), headache (*N* = 31, 24.4%), neuropathic pain syndromes (*N* = 18, 14.2%), back/neck pain (*N* = 5, 3.9%), and gynecological/genitourinary (*N* = 2, 1.6%). The average age at the time of pain disorder diagnosis was 9.24 years (*SD* = 2.41, Range = 4–16). The majority of the sample described onset of first chronic pain symptom in the past three to five years (*N* = 65, 51.2%) and reported last experiencing pain in the past two weeks (*N* = 112, 88.2%).

**Table 1:** General demographic characteristics of youth participants enrolled (*N* = 127)

Demographic characteristic	Percent (%)
Gender	
Males	33.9
Females	66.1
Race	
African American/Black	3.0
Caucasian/White	89.0
Hispanic/Latino	7.0
Ethnicity	
Hispanic/Latino	35.9
Non-Hispanic/Latino	63.8

**Table 2:** General demographic characteristics of parent participants enrolled (*N* = 127)

Demographic characteristic	Percent (%)
Gender	
Males	48.82
Females	51.18
Race	
African American/Black	2.3
Caucasian/White	89.8
Hispanic/Latino	7.0
Ethnicity	
Hispanic/Latino	36.7
Non-Hispanic/Latino	60.2
Education level	
High school graduate, diploma or the equivalent (for example: GED)	3.1
Trade/technical/vocational training	9.4
Associate degree	44.5
Some college credit, no degree	6.3
Bachelor’s degree	32.0
Master’s degree	3.9
Employment status	
Full-time	85.9
Military	0.8
Part-time	10.2
Unable to work	0.8
Unemployed	1.6
Annual income	
$20,000 to $34,999	5.5
$35,000 to $49,999	22.7
$50,000 to $74,999	46.1
$75,000 to $99,999	20.3
Less than $20,000	0.8
Over $100,000	3.9
Marital status	
Divorced	3.1
Married or domestic partnership	93.0
Separated	0.8
Widowed	2.3

### Agreement between child self-report and parent proxy-reported pain-related functional disability

The positive correlation between youth self-report and parent-proxy reported PROMIS functional interference was found to be significant, [r(127) = 0.73, *P* < 0.001]. The youth and parent self-reported score agreement was moderate, met the a priori criterion (minimum) ICC value of 0.5, and was statistically significant (*P* < 0.001).

The group child and adolescent self-reported PROMIS median was 28.54 and the median for the parent-proxy report was 26.33. Means of parent-proxy reports were significantly lower than means of youth self-reports on the PROMIS measure (*p* < 0.05). In other words, parents consistently underestimated their child’s pain-related functional interference.

**Fig. 1:**
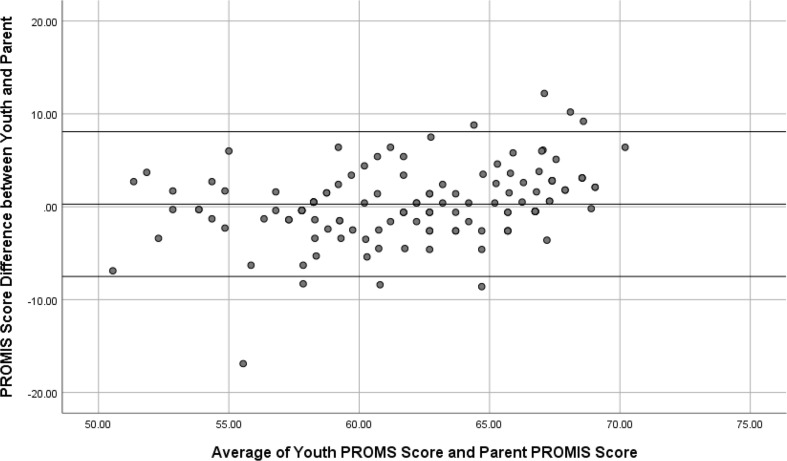
Bland-Altman plot for the PROMIS functional disability scores showing tendency for parent proxy reports to underestimate youth’s pain related functional interference

Bland-Altman plot was used to evaluate any systematic tendency for parent-proxy reports to underestimate child’s pain-related functional disability compared with child’s self-reports of functional disability. The mean youth-parent arithmetic difference was first calculated and no significant difference (*t*(126) = 0.812, *p* = 0.481) was found. A Bland-Altman plot was then generated for the paired child self-report and parent-proxy scores to further assess their agreement. The Bland-Altman plot for the child and parent-proxy reports of PROMIS scores (See Fig. 1) revealed a considerable amount of variation and systematic bias in the child-parent dyad, with 95% limits of agreement, indicating a significant inter-rater variability pattern [*B* = 0.279, *SE* = 0.072, *p* = 0.000].

### Characteristics associated with the difference between child self-report and parent-proxy report PROMIS pediatric scores

Multivariable regression model examined the relationship among the child and adolescent’s age, sex, race, duration of pain, pain intensity, parent pain history and dependent variable of the child-parent agreement on reported pain-related functional interference. There was a statistically significant association between the child and adolescents self-reported pain intensity (β = 0.953, *P* < 0.05) and the difference between the child’s self-reported and parent-proxy reported functional interference scores on the PROMIS. The difference between the child and parent PROMIS scores increased by 0.953 points with each one-point increase in the child and adolescent’s PROMIS score.

## Discussion

The present study examined the child-parent agreement on pain-related functional disability in pediatric chronic pain. The study assessed the level of agreement between child and adolescent’s self-reports and their parent-proxy reports of pain-related functional disability. The agreement between reports of children themselves and their parent-proxy for functional interference was moderate. This is consistent with the literature demonstrating that child-parent agreement among the PROMIS measures were higher for concrete or observable domains, such as pain interference than items measuring internalized states [31–33]. Findings suggest that parents consistently underestimated their child and adolescent’s pain-related functional interference. Existing literature similarly documents cross-informant variance that is often observed in child and adolescent self-reports versus parent-proxy reports of functional outcomes in the context of pediatric pain [20, 23, 34]. This finding further highlights the importance of whose report is most reflective of the child and adolescent’s experience in the context of chronic pain. While difficulties with comprehension of measures questions may pose systemic response biases, research supports children’s ability to understand and respond to the PROMIS questions appropriately [35]. Therefore, child and adolescent comprehension of the PROMIS pediatric measure does not fully explain the noted child self-report and parent-proxy report discrepancy. Findings outline limitations to the extent to which parent-proxy reports offer the most accurate and comprehensive view into the child and adolescents experience with chronic pain. Parents perceive the child’s pain experience through their own viewpoint and their parent-proxy reports of child outcomes should be regarded correspondingly.

Additional insight into the pattern of agreement between child self-reported and parent-proxy reports of pain-related functional disability emerged. A further examination of demographic, medical, and environmental characteristics contributing to the magnitude of differences in child-parent report agreement was completed. The difference between the paired child self-report and parent-proxy report of functional disability was significantly associated with greater child reported pain intensity only. Importantly, findings demonstrate that high pain intensity was associated with greater child-parent rating agreement. These findings are consistent with previous studies of child-parent agreement on functional interference in the context of chronic pain [22, 36].

There are several possible explanations related to child-parent communication patterns about pain-related symptoms, particularly in the setting of mild and moderate child reported pain intensity. Children and adolescents reporting higher levels of pain intensity may be more communicative about how their functioning is impacted in the presence of chronic pain [22, 36]. The functioning of children and adolescents who experience higher levels of chronic pain may also be predictable, less transient, and more recognizable to their parents. It is also possible that parents may respond more readily to child reports of severe pain necessitating medical interventions [20].

An alternative explanation to parental underestimation of pain-related functional interference with lower child-reported pain intensity relates to the lens of internalization of pain experiences. Children with lower pain intensity may present with differing appraisals and internalized concerns in the setting of pain-related functional interference which may be less apparent to their parents [32].

Additional factors associated with the magnitude of difference between child and parent report relate to differing expectation and adaptation to child’s pain experience. Consistent with a phenomenon referred to as recalibration response shift parent’s of children with mild to moderate pain may have conformed with expectations around functional engagement in the context of chronic pain [34, 37]. This may be contrasted with the child and adolescent’s developmentally informed functional expectations which may be determined relative to social comparisons of other healthy similarly aged peers.

Limitations to the study include sampling as the participants enrolled in this study were primarily self-identified as racially White and of higher educational attainment. Since data collection was completed online as well as in-person at pediatric pain clinic, sampling bias embedded in both settings is another study limitation. In addition, children and adolescents participating in this study were aged 8 to 17 years and findings cannot be generalized to younger population. Given the current sample size, examining potential meaningful differences in child-parent agreement on functional disability between mothers and fathers’ dyads requires further research.

## Conclusions

Overall, implications from the current study underscore the lack of complete child-parent agreement on functional disability in the context of pediatric chronic pain. Further understanding of patterns of child-parent agreement informs clinical approaches to the interpretation of parent-proxy reports of chronic pain and related functioning. Relying merely on parent-proxy reports provides a partial perspective on the child and adolescent’s comprehensive pain experience. The study supports that parent-proxy reports should not be viewed as a substitute for child self-reports. During pain management services, health care providers should provide children and adolescents with an equal opportunity to describe their own unique pain experience when developmentally appropriate [2, 38, 39]. Obtaining multiple perspectives in the setting of chronic pain is imperative for optimal patient-centered pain management.

## Data Availability

The datasets used and/or analyzed during the current study are available from the corresponding author on reasonable request.
